# Onset, Duration, and Incidence of Adverse Events by Dose and Titration Schedule of Xanomeline and Trospium Chloride

**DOI:** 10.1192/bjo.2026.11947

**Published:** 2026-06-30

**Authors:** Rachel Dyme, Jenna Hoogerheyde, Ayesha Pavithran, Ranjan Tiwari, James Appio

**Affiliations:** Bristol Myers Squibb, Princeton, USA

## Abstract

**Aims:**

Xanomeline/trospium chloride (KarXT) is approved in the US for schizophrenia in adults. KarXT combines the dual M_1_/M_4_-preferring muscarinic receptor agonist xanomeline with the peripherally restricted pan muscarinic receptor antagonist trospium chloride to reduce side effects related to xanomeline activation of peripheral muscarinic receptors. KarXT efficacy and safety were demonstrated in the EMERGENT trials and a phase 4 trial evaluating tolerability of administration with/without food. This post-hoc analysis compared onset, duration, and incidence of treatment-emergent adverse events (TEAEs) by KarXT dose (100mg/20mg and 125mg/30mg twice daily [BID]) and titration schedule with data from these completed trials.

**Methods:**

The phase 4 “food effect” inpatient, open-label trial (NCT06572449) enrolled adults with schizophrenia, Positive and Negative Symptom Scale (PANSS) total score ≤80, and Clinical Global Impression-Severity (CGI-S) score ≤4. In weeks 1-4, participants began KarXT at 50mg/20mg BID (days 1-7), followed by 100mg/20mg (days 8-14), and increased to a maximum of 125mg/30mg (cohort 1) or continued with 100mg/20mg (cohort 2) (days 15-28). The inpatient, 5-week, randomized, double-blind, placebo-controlled, phase 2EMERGENT-1 (NCT03697252) and phase 3 EMERGENT-2 (NCT04659161) and EMERGENT-3 (NCT04738123) trials enrolled adults with confirmed schizophrenia and recent worsening of psychosis warranting hospitalization, PANSS total score ≥80, and CGI-S score ≥4. KarXT BID dosing began at 50mg/20mg (days 1-2), followed by 100mg/20mg (days 3-7), and increased to a maximum of 125mg/30mg (days 8-35). Food effect trial TEAE analyses by maximum KarXT dose and titration schedule were restricted to weeks 1-4; results from EMERGENT were pooled.

**Results:**

In the food effect trial, KarXT TEAEs occurred in 69.9% (100mg/20mg) and 59.0% (125mg/30mg) of participants; in pooled EMERGENT, rates were 77.8% and 67.1%, respectively. Peak incidence of procholinergic and anticholinergic TEAEs coincided with transition to the KarXT 100mg/20mg dose (food effect: week 2; pooled EMERGENT: days 3-7); incidence of TEAEs plateaued or decreased with transition to KarXT 125mg/30mg. Nausea duration was longer for those maintained on KarXT 100mg/20mg in both datasets. Efficacy was measured by decrease in PANSS total score and observed with 100mg/20mg and 125mg/30mg in both study populations.

**Conclusion:**

Overall, TEAE incidence and duration appeared to be similar regardless of titration speed to KarXT 125mg/30mg BID. Data suggest the duration of certain TEAEs is longer with 100mg/20mg BID, potentially due to the higher ratio of X to T of 5:1 versus 4.1:1. In some circumstances, potential improvements in tolerability may be seen with more rapid titration and/or uptitration to KarXT 125mg/30mg BID.

